# Correlation Between the Water Solubility and Secondary Structure of Tilapia-Soybean Protein Co-Precipitates

**DOI:** 10.3390/molecules24234337

**Published:** 2019-11-27

**Authors:** Li Tan, Pengzhi Hong, Ping Yang, Chunxia Zhou, Dinghao Xiao, Tanjun Zhong

**Affiliations:** 1 College of Food Science and Technology, Guangdong Ocean University, Zhanjiang 524088, China; tanli_food@163.com (L.T.); nhs9701@163.com (P.H.); 50299052@163.com (P.Y.); X107205676@163.com (D.X.); jamiezhong@163.com (T.Z.); 2 Guangdong Provincial Key Laboratory of Aquatic Product Processing and Safety, Zhanjiang 524088, China; 3 Guangdong Provincial Engineering Technology Research Center of Marine Food, Zhanjiang 524088, China; 4 Guangdong Provincial Modern Agricultural Science and Technology Innovation Center, Zhanjiang 524088, China; 5 Southern Marine Science and Engineering Guangdong Laboratory (Zhanjiang), Zhanjiang 524088, China

**Keywords:** tilapia, soybean, protein co-precipitates, protein secondary structure, water solubility, correlation coefficients

## Abstract

The secondary structure of a protein has been identified to be a crucial indicator that governs its water solubility. Tilapia protein isolate (TPI), soybean protein isolate (SPI), and tilapia-soybean protein co-precipitates (TSPC_3:1_, TSPC_2:1_, TSPC_1:1_, TSPC_1:2_, and TSPC_1:3_) were prepared by mixing tilapia meat and soybean meal at different mass ratios. The results demonstrated that the water solubility of TSPCs was significantly greater than that of TPI (*p* <0.05). The changes in ultraviolet–visible and near-ultraviolet circular dichroism spectra indicated that the local structure of TSPCs was different from that of TPI and SPI. Fourier transform infrared Spectroscopy revealed the co-existence of TPI and SPI structures in TSPCs. The secondary structures of TSPCs were predominantly α-helix and β-sheet. TSPC_1:1_ was unique compared to the other TSPCs. In addition, there was a good correlation between the water solubility and secondary structure of TSPCs, in which the correlation coefficients of α-helix and β-sheet were −0.964 (*p* <0.01) and 0.743, respectively. TSPCs displayed lower α-helix contents and higher β-sheet contents compared to TPI, which resulted in a significant increase in their water solubility. Our findings could provide insight into the structure–function relationship of food proteins, thus creating more opportunities to develop innovative applications for mixed proteins.

## 1. Introduction

Tilapia (oreochromis niloticus) is a widely-cultured freshwater fish species in Africa and Asia. This species has attracted a considerable amount of attention due to its low fat levels, high protein contents, being rich in unsaturated fatty acids, and being particularly suitable for children and the elderly [1]. Both tilapia fish meal and tilapia protein isolates, which are low in calories and high in nutritional value, have been successfully introduced into pasta [2,3]. Consumer demand for aquatic foods continues to grow and tilapia proteins have caught the attention of functional food manufacturers [4,5]. However, even though the excellent nutritional properties of tilapia proteins are well-recognized, they remain unable to meet the various needs of food systems because of limitations regarding their water solubility and related functional properties [6,7]. These concerns have triggered numerous manufacturer groups to develop new ways to solve the problem. Recently, animal proteins have been combined with plant proteins to improve the quality of food products [8]. Several studies have combined both animal and plant proteins to obtain a material with excellent processing properties for food industry applications [9,10].

Among the known vegetable proteins, soybean protein represents the largest source of plant-based protein in the world. Soybean protein has a wide range of applications in infant formulas due to its good solubility and high nutritional value [11,12]. Numerous studies have confirmed that soybean protein is suitable for mixed protein systems, including soy globulins and β-lactoglobulin [13], soy protein and micellar caseins [14], and soy protein and bovine collagen [15]. In most cases, mixed protein systems exhibit excellent gelling, film formation, and other surface functional properties during their application in the food industry [8,16]. More recently, soy protein and cod protein were integrated to construct a double protein network that is expected to find many applications in the food and other relevant industries [17]. With the ever-increasing demand for novel food products, systems that mix fish proteins and plant proteins are worth further investigation and development [16]. Therefore, combining tilapia protein with soy protein may be considered an ideal way to achieve the protein properties that food manufacturers require.

Protein co-precipitates are common protein complexes in mixed protein systems [18]. Co-precipitation can serve as an excellent strategy for mixing tilapia protein and soybean protein. Existing studies suggest that whey-soy protein co-precipitates, which contain the protein components of whey protein and soybean protein, exhibit a high water-holding capacity and good gelling properties [19]. Sheep milk protein co-precipitates were found to have a high foaming capacity and good stability at all pH values [20]. Additionally, the nutritional properties of protein co-precipitates can be enhanced by altering their amino acid compositions [21]. A recent study showed that protein co-precipitates, as a food ingredient, can provide angiotensin-converting enzyme (ACE) inhibitory activity and antioxidant activities for high-selectivity applications [22]. The combination of a wide range of physical, functional, and nutritional properties allows for protein co-precipitates to exhibit the desired processing properties and be suitably used as an ingredient in various food systems.

In a mixed protein system, the structural diversity and amphiphilicity of the food proteins allow them to interact with other proteins in food products under certain conditions, which may cause structural changes to occur in the food proteins and affect the functional properties of the food products [8,13]. To better understand the relationship between structural changes and functional properties, numerous studies have used spectral analyses to determine the structural alterations in mixed protein systems. For instance, the formation of a rice protein and whey protein complex has been verified by both fluorescence and ultraviolet-visible (UV-Vis) spectra [23]. Fourier Transform Infrared (FTIR) spectral data have revealed that casein non-phosphopeptides and soybean polypeptides can be aggregated to generate new chemical bonds [24]. Through near-ultraviolet circular dichroism (near-UV CD) and far-UV CD studies, it was found that rice protein can be integrated into soy protein to form more complicated structures in order to improve its water solubility [25,26]. Therefore, a systematic and thorough study on the structural changes in a mixed tilapia protein and soybean protein system is required in order to evaluate its functional properties.

It is well known that the secondary structure of a protein is the basis for its properties in a food system. During processing, the a-helix was shown to be negatively related to the elastic modulus of silver carp myosin and the gel strength of shrimp surimi, while the β-sheet was shown to be positively correlated [27,28]. It has been reported that the β-sheet and β-turn content in silver carp myosin increased after glycation, suggesting that a highly flexible conformation is important for determining its functional properties [29]. These correlation studies have demonstrated that the structural flexibility of a protein can provide us with a deep understanding of its functional properties. Moreover, the water solubility of a food protein plays a significant role in its functional properties, such as foaming, emulsifying, and gelling, and serves as a protein alteration index during food processing [30,31]. In addition, food proteins with excellent water solubility have been shown to be promising interfacial materials. Several studies have discussed the importance of the water solubility of mixed protein systems in food industry applications [25,26]. A good correlation coefficient (0.920, *p* <0.01) was found between the molecular flexibility and emulsifying properties of food proteins [32]. However, the correlation between the secondary structure and water solubility of proteins has rarely been reported on. Therefore, to broaden the range of applications of mixed tilapia protein and soybean protein systems, it is necessary to explore the relationship between the secondary structure and water solubility of these proteins.

In the present study, tilapia-soybean protein co-precipitates (TSPCs) were prepared by mixing tilapia meat and soybean meal at different mass ratios. The water solubility and structural characteristics of the TSPCs were determined, and in this respect, the importance of secondary structures is discussed. In addition, other relevant structural features of TSPCs were analyzed at different levels. Our findings could expand the potential ways to prepare novel mixed protein systems and help to provide a theoretical framework for the structure–function analysis of protein co-precipitates.

## 2. Results and Discussion

### 2.1. Analysis of the Water Solubility of TSPCs

**Figure 1:**
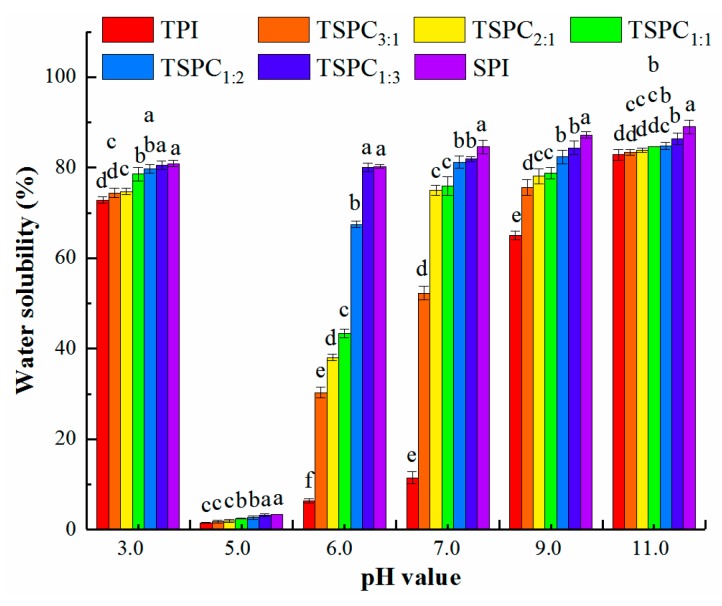
Water solubility of tilapia protein isolate (TPI), tilapia-soybean protein co-precipitates (TSPCs), and soybean protein isolate (SPI). Different letters with the same pH value indicate significant differences in the water solubility of the samples (*p* < 0.05).

The water solubility of proteins can affect their functional performances when applied in beverages and infant formula, and operates as an index of structural alteration during food processing [31]. Figure 1 illustrates the solubility patterns of the tilapia protein isolate (TPI), TSPCs, and soybean protein isolate (SPI) that were obtained from the pH shift process. The pH-solubility profiles of the TSPCs are U-shaped, which is similar to a previous study on water solubility, in a wide pH range of tilapia protein concentrate [7] and commercial soy proteins [11]. Typically, the water solubility at various pH values is used to indicate the performance of protein co-precipitates when they are incorporated into food systems. As shown in Figure 1, the water solubility of the TSPCs was significantly greater than that of the TPI (*p* <0.05), which is consistent with previous findings about the higher water solubility of soybean-whey co-precipitates than that of separate protein co-precipitates [33]. The greatest changes (an increase of more than 40%) in solubility values between the TPI and the TSPCs occurred at pH 7.0. At a neutral pH, TSPC_1:3_ displayed the highest water solubility of 81.9%, indicating that TSPCs are appropriate for use in food formulations. Therefore, TSPCs can serve as ingredients that help to achieve excellent functional properties in a variety of foods.

However, the water solubility value of TSPC_1:1_ was not significantly different compared to that of TSPC_2:1_ at pH 7.0 and 9.0, as well as that of the other TSPCs at pH 11.0. The changes in the water solubility of the TSPCs can be explained in terms of two aspects: (i) proteins have either a net positive or a net negative charge at low and high pH values, leading to an electrostatic repulsive force that separated the protein molecules and further increased their water solubility [34]; and (ii) the molecular structures of the TSPCs may have extended and become less tense, which enabled them to easily spread out in the water, resulting in the different values of water solubility. Existing research suggests that the solubility of proteins is dependent on its structure, the pH, and other intrinsic factors [35]. To elucidate the mechanism that underlies this phenomenon, the structural characteristics of the TSPCs were further assessed using different spectral experiments.

### 2.2. Analysis of the TSPCs’ UV-Vis Spectra

**Figure 2:**
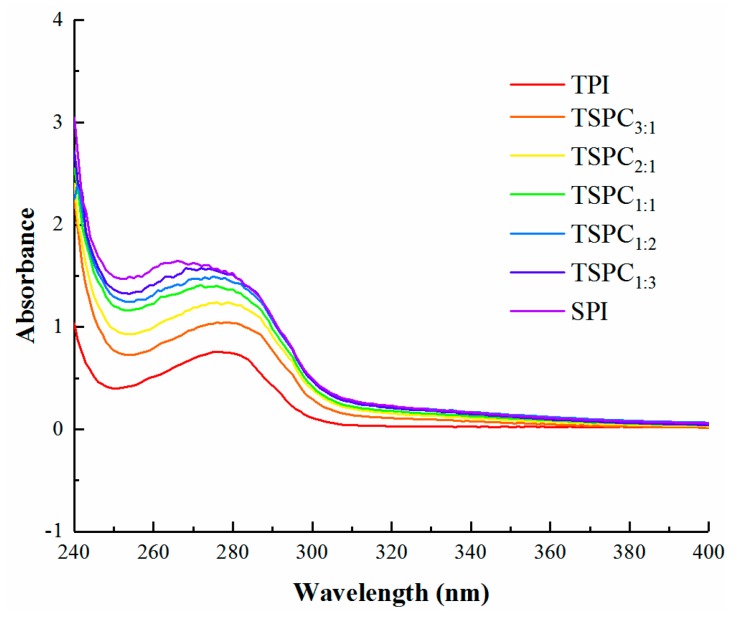
Ultraviolet-visible (UV-Vis) spectra of the TPI, TSPCs, and SPI.

UV-Vis spectra of protein solutions are usually sensitive to protein conformations [36]. As shown in Figure 2, the TSPCs displayed similar peaks of aromatic amino acids within the range of 260–280 nm and the absorbance values (at 280 nm) of the TSPCs were found to gradually increase. This result is probably due to the π-π* transition of the benzene rings of tryptophan and tyrosine on the protein peptide chains. Such increased UV-Vis absorption intensities reflect the weakening of the hydrogen-bond interactions in the TSPCs molecules compared to the TPI and indicate the extension of peptide chains, which can influence the molecular structure of TSPCs. Moreover, the UV-Vis absorption peaks of the TSPCs exhibited a tendency to move toward a short wavelength. In comparison with the TPI, the absorption peaks of TSPC_1:1_, TSPC_1:2_, and TSPC_1:3_ shifted slightly to a shorter wavelength. Previous studies have confirmed that crosslinking of peptide chains occurs between rice protein and whey protein under strongly alkaline conditions, which is caused by hydrogen bonds, hydrophobic forces, and electrostatic interactions and leads to changes in the aromatic amino acids [23]. Regarding the tilapia and soybean co-solved at pH 11.0, the peptide chains of the two original proteins may also have been cross-linked, which subsequently altered the molecular structure of the TSPCs and led to the alterations in the UV spectra. Thus, through the responses to aromatic amino acids, the changes in the local structure of the TSPCs were determined by analysis of UV-Vis spectra.

### 2.3. Near-UV CD Analysis of the TSPCs

**Figure 3:**
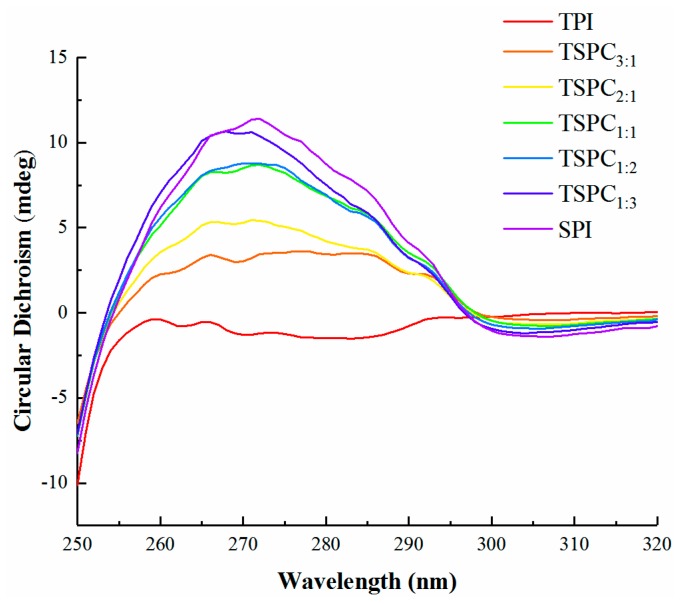
Near-UV CD spectra of the TPI, TSPCs, and SPI.

CD spectra are widely used to analyze the structure of proteins [37]. A near-UV CD signal is derived from the chirality of the side chain environment of amino acid residues and can reflect the molecular structure of proteins as depicted by the interaction and orientation of the aromatic ring of its tyrosine, tryptophan, and phenylalanine with other amino acid moieties [25,38]. Each aromatic amino acid tends to display a typical wavelength profile in near-UV CD spectra [39]. As shown in Figure 3, the TPI and TSPC_3:1_ exhibited tyrosine peaks between 275 and 282 nm, while the SPI and the other TSPCs displayed phenylalanine peaks between 255 and 270 nm. These findings indicate that the amino acid residues of the TSPCs side chains were different from those of tilapia protein and soybean protein. Moreover, the tyrosine and phenylalanine in the TSPCs was demonstrated to have a prominent positive ellipticity band with an upward shift. The near-UV CD spectra of the protein samples exhibit opposite signals, which may indicate chiral inversion [40]. It was found that the molar ellipticity of TSPCs was higher than that of the TPI, indicating stronger interactions among the aromatic residues. Generally, the order of the arrangement of the local amino acids as well as the interactions between proteins and other molecules can affect the secondary structure of proteins [25,41]. Besides this, the hydrogen bonds between amino hydrogen and carbonyl oxygen play a critical role in maintaining the α-helix structure [42,43]. The changes in the absorption peaks of the near-UV CD spectra revealed that the TSPCs may have had less tense structures compared to the TPI. Consistent findings have been reported on the ellipticity values of seed flour proteins that are associated with the interactions between aromatic residues, in which a stronger interaction causes a lower proportion of α-helix structure in seed flour proteins [44].

Based on the abovementioned spectral analysis, compared to the TPI and SPI, changes in the molecular structures of the TSPCs were observed, which could dramatically affect their water solubility values. However, UV-Vis and near-UV CD spectra only reflect the local protein structure of TSPCs. To identify the cause of the changes in the water solubility, a comprehensive analysis was performed on the secondary structure of the TSPCs.

### 2.4. FTIR Spectral Analysis of the TSPCs

**Figure 4:**
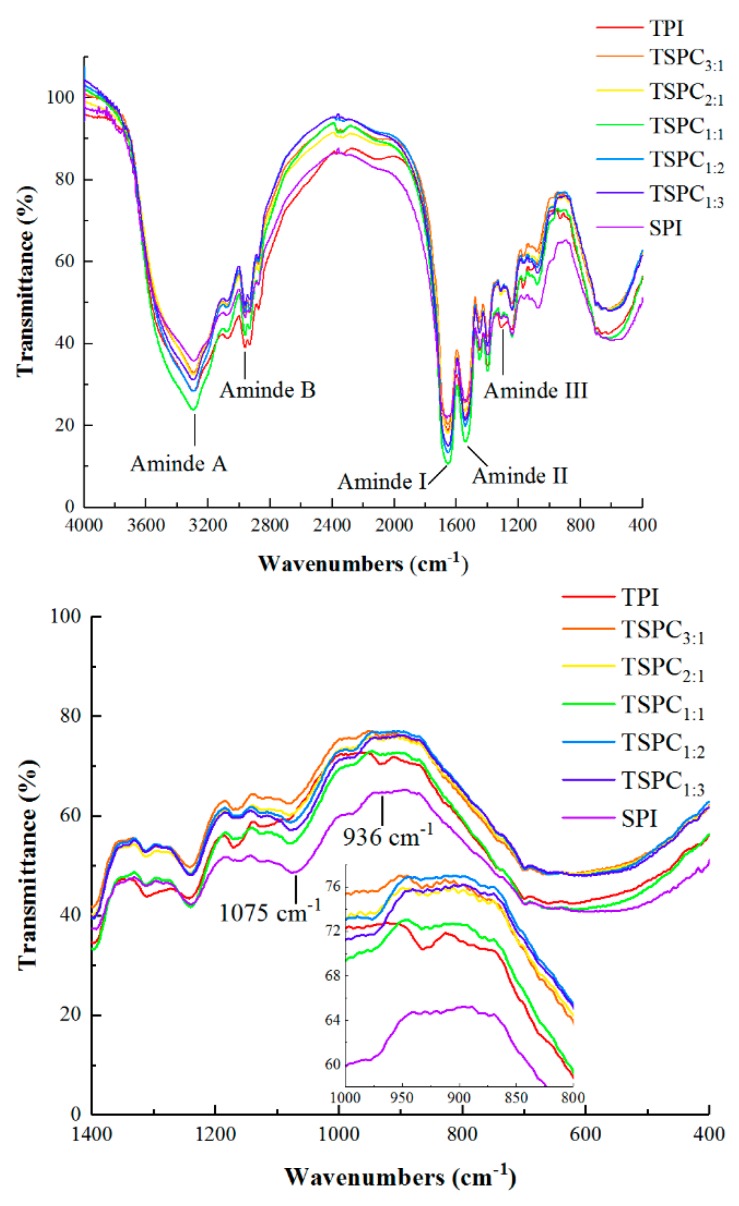
FTIR spectra of the TPI, TSPCs, and SPI.

FTIR spectroscopy is a valuable method for monitoring the changes in the secondary structure of proteins [45]. As shown in Figure 4, the FTIR spectra of the TSPCs within the range of 400–4000 cm^−1^ showed that the characteristic adsorption band was similar to those observed in the proteins that were isolated from tilapia and soybean. It is worth noting that the amide A band, amide B band, amide I band, amide II band, amide III band, and several adsorption peak areas (400–800 cm^−1^) are the characteristic group frequencies that are produced by the repetitive polypeptide units of proteins [46]. The absorption band of the TSPCs at the amide A band was attributed to the stretching of N-H, and the amide B band was attributed to changes in the C-H stretching. The amide I band was associated with C=O stretching, the amide II band was affected by C-N stretching, and the amide III band was related to changes in C-N stretching and N-H bending [32]. Additionally, several adsorption peaks within the range of 400–800 cm^−1^ were attributed to the vibrations of C-H and N-H bonds in the TSPCs.

According to the obtained FTIR spectra data, the absorption intensities of the TSPCs were significantly different from those of the TPI and the SPI. Regarding the infrared spectrum of the amide A, amide I, and amide II bands, the infrared absorption intensity of the TSPCs was markedly weaker than that of the TPI and there was an obvious displacement at the amide B and amide III bands. These results indicate that the intramolecular and intermolecular hydrogen bonds of the TSPCs were weaker than those of the TPI (Figure 2). Moreover, the TSPCs demonstrated an absorption peak at 1075 cm^−1^, which was found in the SPI, but was not observed in the TPI. The protein structures of TSPC_3:1_, TSPC_2:1_, and TSPC_1:1_ were found to be similar to those of the TPI, in which an absorption peak was observed at 936 cm^−1^. These results prove that the tilapia protein and soybean protein structures co-existed in the TSPCs. This conclusion is supported by the fact that a soybean polypeptide and casein non-phosphopeptide protein complex was found to contain two protein structures [24]. As presented in Figure S1 and the Supplementary Materials, the TSPCs contained mixed subunit components of TPI and SPI, further verifying the co-existence of tilapia protein and soybean protein structures in the TSPCs. Additionally, TSPC_1:1_ exhibited a weaker absorption intensity compared to the other TSPCs, indicating that it may possess unique protein structures. To further explore the structural variability among the five TSPCs, the contents of their secondary structures were analyzed.

### 2.5. Determination of the Secondary Structure of the TSPCs

**Figure 5:**
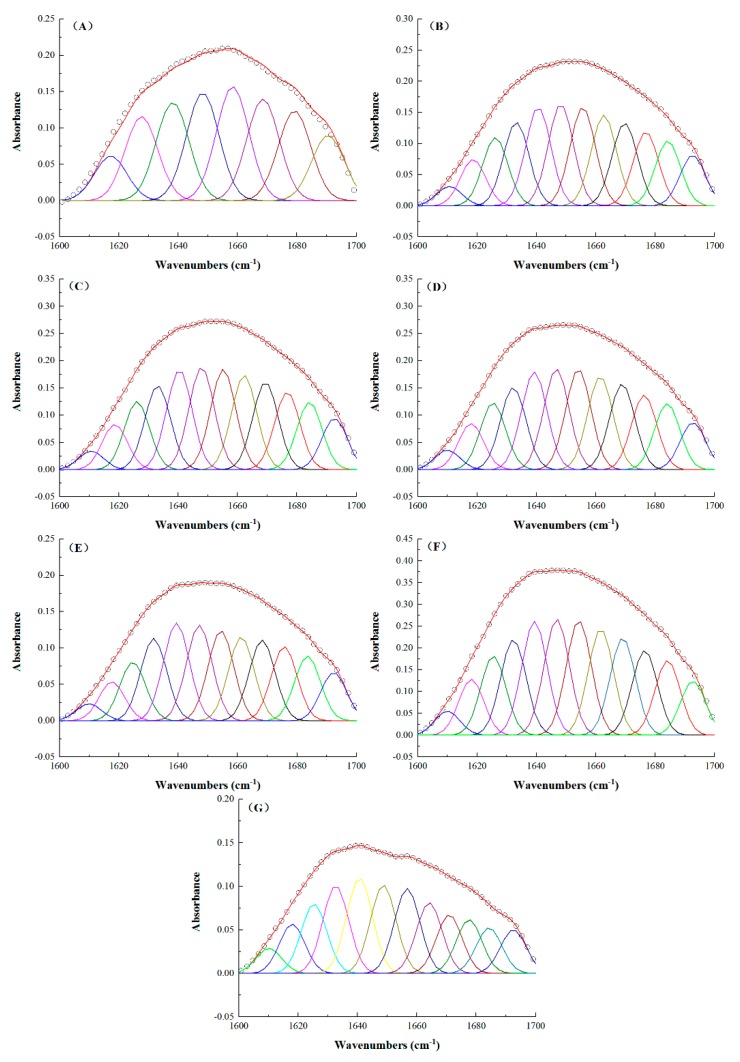
Infrared spectra of the amide I bands of the TPI (**A**), TSPC_3:1_ (**B**), TSPC_2:1_ (**C**), TSPC_1:1_ (**D**), TSPC_1:2_ (**E**), TSPC_1:3_ (**F**), and the SPI (**G**).

To further analyze the contents of the secondary structures in the TSPCs, the infrared spectra of the amide I band at 1600–1700 cm^−1^ were fitted using Peak Fit software. The correspondence relationships of the secondary structure were as follows: 1645–1660 cm^−1^ (α-helix), 1610–1637 cm^−1^ and 1670–1690 cm^−1^ (β-sheet), 1660–1670 cm^−1^ (β-turn), and 1637–1645 cm^−1^ (random coil) [45,47]. As shown in Figure 5, a few fitting peaks of TPI were observed at the amide I band. However, similar to the SPI, the TSPCs exhibited an abundance of fitting peaks. The relative contents of the secondary structures in the TSPCs were calculated according to the integrated area of the fitting data of the amide I band.

The secondary structures of the TSPCs as analyzed by FTIR spectroscopy are presented in Table 1. Notably, the TPI exhibited the highest content of α-helix, while the SPI displayed the highest content of β-sheet. These results are in agreement with previous studies demonstrating that the α-helix content in TPI is higher than that in SPI [29,47]. As shown in Table 1, the TSPCs contained two main structures: α-helix and β-sheet. It was observed that the α-helix content was significantly decreased in the TSPCs compared to the TPI, while the β-sheet content was significantly increased. Moreover, Figure 2 and Figure 4 illustrate the changes in the absorption intensities of the TSPCs as well as the weakened intramolecular and intermolecular hydrogen bonds in TSPCs, which confirm the decrease in the α-helix content in the TSPCs compared with the TPI. Compared to the TPI and the SPI, the TSPCs exhibited the highest β-turn and the lowest random coil, indicating that the secondary structures of the TSPCs are significantly different from those of the TPI and the SPI. The reason for the differences in contents might be that tilapia protein and soybean protein extend their molecular structure under alkaline conditions, causing depolymerization and rearrangement of the protein subunits. These results agree well with previous observations that the two protein components are integrated into a novel protein complex [25,48].

In addition, TSPC_1:1_ exhibited higher β-turn content and lower β-sheet and random coil contents, as compared to the other TSPCs, suggesting that TSPC_1:1_ contains a unique protein structure. These results are supported by the FTIR spectral data, where TSPC_1:1_ was found to have the lowest absorption intensity. As shown in Figure 1, at pH 7.0 and 9.0, the water solubility of TSPC_1:1_ was not significantly increased compared to TSPC_2:1_. These results may have been produced by the combined effect of the increased β-turn content and decreased α-helix, β-sheet, and random coil contents in TSPC_1:1_. Additionally, the water solubility of the TSPCs was found to be better than that of the TPI, which also resulted from the combined effect of the various secondary structures. These findings are similar to those of previous work, which found that both α-helix unfolding and β-sheet formation favor the gelation of myosin [28,43]. According to previous studies [29,32,49], flexible structures are conducive to function-related conformational changes, while tight structures may not be beneficial to functional enhancement. In this study, the tight structure of tilapia proteins may have partially contributed to their highly flexible structures, thus improving the water solubility of the TSPCs. However, the order of the changes in the functional properties of the TSPCs was not completely consistent with their secondary structures. Therefore, the correlation between water solubility and the different secondary structures of the TSPCs was further analyzed.

### 2.6. The Correlation Between the Water Solubility and Secondary Structure of the TSPCs

The correlation between water solubility (at pH 6.0, 7.0, and 9.0) and secondary structure (including α-helix, β-sheet, β-turn, and random coil) was analyzed by Pearson’s correlation coefficients using SPSS software version 19.0. The correlation coefficient values of the TPI, SPI, and TSPCs between water solubility and secondary structure are presented in Table 2. For different secondary structures, the correlation coefficients of α-helix and β-sheet with water solubility demonstrated a higher level of comparison. It was found that the α-helix contents of the TSPCs were significantly negatively correlated with their water solubility (R = −0.964, *p* <0.01). On the contrary, the β-sheet contents of the TSPCs were positively correlated with their water solubility (R = 0.743). These results are similar to those of previous work, which found that the a-helix was negatively related to the gel strength, while the β-sheet showed a positive correlation [27,28]. Under pH 6.0 and 9.0 conditions, a positive correlation between the β-sheet content and water solubility of TSPCs was observed (R = 0.863, *p* <0.05). This result may be attributed to the changes in the protein’s secondary structure after pH adjustment [44]. However, the pH changes did not affect the positive or negative correlation between water solubility and secondary structure (for α-helix and β-sheet, respectively). Such excellent correlation results indicate that the secondary structure of a protein is an important indicator of its water solubility. In addition, the effect of α-helix on water solubility was found to be greater compared to the other secondary structures. Therefore, it can be speculated that the enhanced water solubility of TSPCs is mainly influenced by the lower contents of α-helix in comparison with TPI. This phenomenon would be consistent with prior research, which found that α-helix displays a tight structure with no cavities and may be detrimental to the specific conformational change that is required for the emulsifying properties of SPI [50]. Besides this, compared to TSPC_2:1_, the water solubility of TSPC_1:1_ was not significantly increased (Figure 1), which could be due to its lower β-sheet content. Taken together, our results demonstrate that the secondary structure of a food protein is closely correlated to its water solubility, and both α-helix and β-sheet structures were identified as the main correlation factors.

## 3. Materials and Methods

### 3.1. Materials

Fresh Nile tilapias (Oreochromis niloticus) were purchased from a local fish market in Zhanjiang, Guangdong, People’s Republic of China. After being cryogenically frozen and transported back to the laboratory, fish muscle with a protein content of 17.73% (N × 6.25, wet) was carefully collected and stored at −20 °C until analysis. Soybean meal with a protein content of 53.31% (N × 6.25, wet) was purchased from Shandong Wandefu Biotechnology Co., Ltd. (Dongying, China). All chemicals that were used were of analytical grade and were used without further purification.

### 3.2. Preparation of Tilapia-Soybean Protein Co-Precipitates

Tilapia-soybean protein co-precipitates (TSPCs) were prepared according to a previously described method with some modifications [19]. Tilapia meat and soybean meal (tilapia fish:soybean meal = 3:1, 2:1, 1:1, 1:2, and 1:3) were mixed at a 1:9 (*w/v*) ratio in cold deionized water. The mixtures were adjusted to pH 11.0 with 2.0 M NaOH, followed by stirring for 30 min, and centrifuged at 10,000× *g* for 20 min at 4 °C. The pH of each supernatant was adjusted to 4.5 with 2.0 M HCl and then was centrifuged at 10,000× *g* for 20 min at 4 °C. The pH value of 4.5 was selected based on our preliminary study (Figure S2 in the Supplementary Materials). The resulting precipitates were washed with deionized water and adjusted to pH 7.0 before analysis and then freeze-dried to obtain TSPCs. The TSPCs with a protein content of ≥ 90.0% (N × 6.25, wet) were denoted TSPC_3:1_, TSPC_2:1_, TSPC_1:1_, TSPC_1:2_, and TSPC_1:3_ (Table S1 in Supplementary Materials). The preparation of tilapia protein isolate (TPI) from tilapia meat was carried out as described previously [30], except that the pH of the supernatants was adjusted to 5.5. The obtained TPI displayed a protein content of 95.93% (N × 6.25, wet). The preparation of soybean protein isolate (SPI) from soybean meal was performed as described previously [12], with the exception that the homogenates were adjusted to pH 8.0 and the pH of the supernatants was adjusted to 4.5. The resulting SPI exhibited a protein content of 90.68% (N × 6.25, wet). All protein samples were stored at −20 °C until further analysis.

### 3.3. Determination of Water Solubility

The water solubility of the protein samples was determined according to a previously described method with some modifications [30]. TSPCs powders (300 mg) were dispersed in 30 mL of deionized water. The mixtures were adjusted to pH 3.0 and up to pH 11.0 with 1.0 M HCl or 1.0 M NaOH, respectively, and then stirred for 30 min. After centrifuging at 10,000× *g* for 20 min, the supernatants were collected and analyzed by the Lowry method using an ultraviolet spectrometer (Cary 60, Agilent Ltd., Santa Clara, CA, USA) [51]. The water solubility of each protein sample was calculated according to the following equation:

(1) $$\mathrm{Water}\;\mathrm{solubility}\;(\%)=\frac{\mathrm{Supernatant}\;\mathrm{protein}\;\mathrm{content}}{\mathrm{Solution}\;\mathrm{protein}\;\mathrm{content}\;\mathrm{before}\;\mathrm{centrifugation}}\times 100\%.$$

### 3.4. Ultraviolet-Visible (UV-Vis) Spectroscopy

A specific amount of freeze-dried TSPCs powder was collected and prepared as a 1.0% (*w/v*) protein stock solution at pH 7.0. The stock solution was then centrifuged at 10,000× *g* for 20 min. After evaluating the supernatant protein contents by the Lowry method [51], the stock solution was diluted into a 1.0 mg/mL protein solution. For UV-Vis spectroscopic analysis, deionized water was used as a blank control and the ultraviolet spectra (Cary 60 UV-Vis, Agilent Ltd., Santa Clara, CA, USA) were measured within the range of 240–400 nm.

### 3.5. Near-Ultraviolet Circular Dichroism (Near-UV CD)

The near-UV CD of TSPCs was determined by a CD spectrometer (CHIRASCAN, Applied Photophysics Ltd., Leatherhead, UK) using a previously described method with some modifications [25]. A specific amount of freeze-dried TSPCs powder was collected and prepared as a 1.0% (*w/v*) protein stock solution at pH 7.0. The stock solution was then centrifuged at 10,000× *g* for 20 min. Subsequently, the protein contents of the resulting supernatants were assessed using the Lowry method [51], followed by dilution into a 1.0 mg/mL protein solution. Near-UV CD measurements were recorded with a 10-mm cuvette and the data are expressed in terms of molecular ellipticity.

### 3.6. Fourier Transform Infrared (FTIR) Spectroscopy

Freeze-dried TSPCs powders and potassium bromide were mixed at a ratio of 1:100 and then ground. Plain potassium bromide was used as a blank control group. The protein samples were scanned 16 times at a resolution of 4 cm^−1^ and the infrared spectra (TENSOR27, BRUKER Ltd., Ettlingen, Germany) were recorded within the range of 400–4000 cm^−1^. The obtained infrared spectra were subjected to fitting analysis using Peak Fit software (version 4.12, Systat Software, Inc., Richmond, VA, USA) and a multipeak fitting program with Gaussian deconvolution. The amide I band at 1600–1700 cm^−1^ was analyzed in order to calculate the ratio of the secondary structure of the protein samples.

### 3.7. Statistical Analysis

All experimental measurements were performed in parallel at least three times. Statistical differences between the groups were compared by one-way analysis of variance (AVONA) and Duncan’s Multiple Range test using SPSS software version 19.0 (SPSS Inc., Chicago, IL, USA). Based on the normality test results, Pearson’s correlation method was used to determine the relationship between each variable. All data are expressed as mean ± standard deviations (SD), unless specifically mentioned. A *p* value of less than 0.05 was considered to be statistically significant.

## 4. Conclusions

The isoelectric solubilization/precipitation method was shown to be effective in preparing TSCPs. It was found that the protein co-precipitates displayed excellent water solubility for the purpose of food industry applications. Notably, TSPC_1:3_ exhibited the highest water solubility of 81.9% at a neutral pH. The structural properties of the TSPCs were further characterized to probe the relationship between their water solubility and secondary structure. The absorption intensities and displacements of the UV-Vis spectra and near-UV CD spectra indicate that the local structure of the TSPCs was different from that of the TPI and SPI. Moreover, FTIR spectra revealed the whole protein structure of each TSPC, which verified the co-existence of tilapia protein and soybean protein structures in the TSPCs. The secondary structures of the TSPCs were predominantly α-helix and β-sheet, and TSPC_1:1_ was unique compared to the other TSPCs. The results of the correlation analysis showed a good correlation between water solubility and secondary structure, in which the correlation coefficients of α-helix and β-sheet were −0.964 (*p* < 0.01) and 0.743, respectively. The TSPCs exhibited lower proportions of α-helix and higher proportions of β-sheet compared to the TPI, which significantly (*p* < 0.05) improved their water solubility. In addition, compared to TSPC_2:1_, TSPC_1:1_ displayed a slightly lower β-sheet content, which resulted in an increase in its water solubility; however, the difference was not statistically significant (*p* > 0.05). These findings indicate that the secondary structure of a food protein is closely correlated with its water solubility, and a tight structure is not appropriate for the enhancement of functional properties. Although a proteomics strategy could be used to further analyze the protein structures of the five TSPCs, we consider the relationship between their water solubility and secondary structure to have been demonstrated.

In this paper, the secondary structures of TSPCs were introduced into an analysis of their functional properties. This systematic study on the correlation between the water solubility and secondary structure of TSPCs could provide us with a thorough understanding of and theoretical basis for further study of protein co-precipitates. In conclusion, the structure–function relationship provides important information about food proteins and can serve as a useful index to evaluate the applicability of protein co-precipitates in the food industry.

## Figures and Tables

**Table 1:** The secondary structure contents of the TPI, TSPCs, and SPI.

Items	α-Helix	β-Sheet	Β-Turn	Random Coil
TPI	32.09 ± 0.60 ^a^	39.59 ± 0.62 ^d^	14.86 ± 0.47 ^d^	13.39 ± 0.54 ^a^
TSPC_3:1_	23.71 ± 0.93 ^b^	45.59 ± 0.40 ^bc^	20.60 ± 0.86 ^a^	11.53 ± 0.33 ^b^
TSPC_2:1_	22.43 ± 0.42 ^c^	45.84 ± 0.41 ^bc^	20.37 ± 0.08 ^ab^	11.40 ± 0.28 ^b^
TSPC_1:1_	22.24 ± 0.48 ^c^	44.96 ± 0.74 ^c^	21.18 ± 0.85 ^a^	11.28 ± 0.16 ^b^
TSPC_1:2_	22.30 ± 0.13 ^c^	46.19 ± 0.10 ^b^	19.20 ± 0.57 ^c^	11.64 ± 0.14 ^b^
TSPC_1:3_	22.15 ± 0.45 ^c^	46.03 ± 0.01 ^b^	19.31 ± 0.73 ^bc^	11.83 ± 0.62 ^b^
SPI	22.02 ± 0.35 ^c^	54.04 ± 0.80 ^a^	10.23 ± 0.06 ^e^	13.05 ± 0.69 ^a^

Different letters in the same column indicate significant differences in the secondary structure of samples (*p* < 0.05).

**Table 2:** Analysis of the correlation between the water solubility and secondary structure of TSPCs.

Water Solubility	α-Helix	β-Sheet	β-Turn	Random Coil
pH 6.0	−0.757 *	0.776 *	−0.209	−0.135
pH 7.0	−0.964 **	0.743	0.131	−0.527
pH 9.0	−0.891 **	0.863 *	−0.120	−0.288

*: *p* < 0.05; **: *p* < 0.01.

## Supplementary Materials

The following are available online, Figure S1: SDS-PAGE of tilapia protein isolate (TPI), tilapia-soybean protein co-precipitates (TSPCs), and soybean protein isolate (SPI), Figure S2: Comparison of soluble protein concentration of tilapia protein isolate (TPI), tilapia-soybean protein co-precipitates (TSPCs), and soybean protein isolate (SPI) at different pH value, Table S1: Protein content (g/100g) of tilapia protein isolate (TPI), tilapia-soybean protein co-precipitates (TSPCs), and soybean protein isolate (SPI).
