# Supplementary material for: Correlation Between the Water Solubility and Secondary Structure of Tilapia-Soybean Protein Co-Precipitates
Source: Molecules. 2019 Nov 27;24(23):4337. doi: 10.3390/molecules24234337 (PMC6930460; doi:10.3390/molecules24234337)
Supplement: Supplementary file 1 [file molecules-24-04337-s001.pdf]

Supporting materials

Correlation Between the Water solubility and Secondary Structure of Tilapia-Soybean Protein Co-Precipitates

Li Tan [1,2,3,4], Pengzhi Hong [1,2,3,4,5], Ping Yang [1,2,3,4], Chunxia Zhou [1,2,3,4,5,*], Dinghao Xiao [1,2,3,4] and Tanjun Zhong [1,2,3,4]

1 College of Food Science and Technology, Guangdong Ocean University, Zhanjiang 524088, China; tanli_food@163.com (L.T.); nhs9701@163.com (P.H.); 50299052@163.com (P.Y.); X107205676@163.com (D.X.); jamiezhong@163.com (T.Z.)
2 Guangdong Provincial Key Laboratory of Aquatic Product Processing and Safety, Zhanjiang 524088, China
3 Guangdong Provincial Engineering Technology Research Center of Marine Food, Zhanjiang 524088, China
4 Guangdong Provincial Modern Agricultural Science and Technology Innovation Center, Zhanjiang 524088, China
5 Southern Marine Science and Engineering Guangdong Laboratory (Zhanjiang), Zhanjiang 524088, China
* Correspondence: chunxia.zhou@163.com (C.Z.); Tel.: +86-13828262885

Supporting Figures

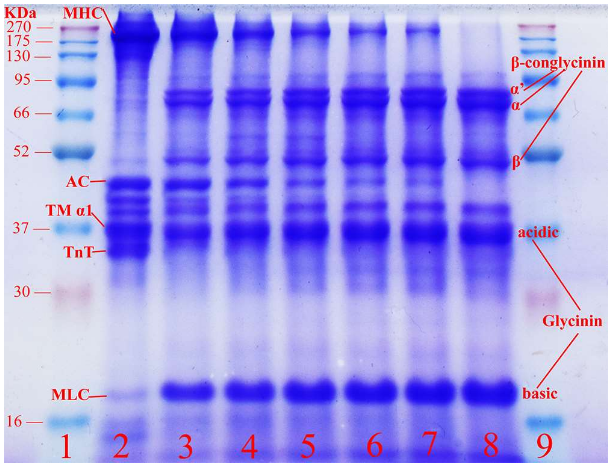

Figure S1. SDS-PAGE of tilapia protein isolate (TPI), tilapia-soybean protein co-precipitates (TSPCs), and soybean protein isolate (SPI).

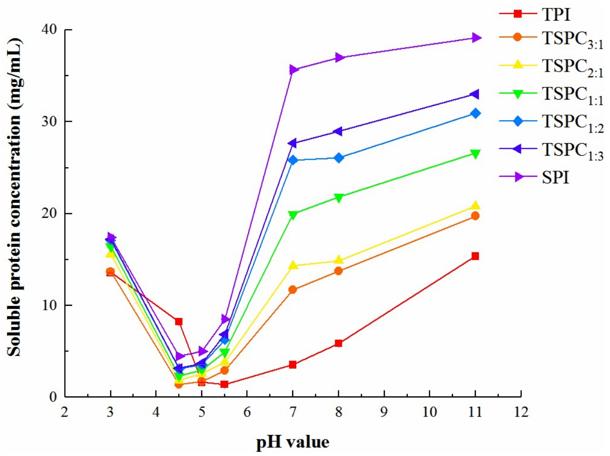

Figure S2. Comparison of soluble protein concentration of tilapia protein isolate (TPI), tilapia-soybean protein co-precipitates (TSPCs), and soybean protein isolate (SPI) at different pH value.

Supporting Table

Table S1. Protein content (g/100g) of tilapia protein isolate (TPI), tilapia-soybean protein co-precipitates (TSPCs), and soybean protein isolate (SPI).

Items	TPI	$TSPC_{3:1}$	$TSPC_{2:1}$	$TSPC_{1:1}$	$TSPC_{1:2}$	$TSPC_{1:3}$	SPI
Protein content	95.93 ± 0.72^{a}	94.24 ± 0.57^{b}	92.76 ± 0.83^{c}	92.67 ± 0.51^{c}	92.05 ± 0.74^{c}	90.84 ± 0.13^{d}	90.68 ± 0.32^{d}

Different letters indicate significant differences (p <0.05).
